# Dietary Enrichment with 20% Fish Oil Decreases Mucus Production and the Inflammatory Response in Mice with Ovalbumin-Induced Allergic Lung Inflammation

**DOI:** 10.1371/journal.pone.0163819

**Published:** 2016-09-26

**Authors:** Jean A. Hall, Jaye Hartman, Monica M. Skinner, Adam R. Schwindt, Kay A. Fischer, William R. Vorachek, Gerd Bobe, Beth A. Valentine

**Affiliations:** 1 Department of Biomedical Sciences, College of Veterinary Medicine, Oregon State University, Corvallis, OR, United States of America; 2 Department of Microbiology, College of Science and College of Agricultural Sciences, Oregon State University, Corvallis, OR, United States of America; 3 Department of Animal and Rangeland Sciences, College of Agricultural Sciences, Oregon State University, Corvallis, OR, United States of America; 4 Linus Pauling Institute, Oregon State University, Corvallis, OR, United States of America; Universidade do Estado do Rio de Janeiro, BRAZIL

## Abstract

The prevalence of asthma has increased in recent decades, which may be related to higher dietary intake of (n-6) polyunsaturated fatty acids (PUFA) and lower intake of (n-3) PUFA, e.g., those contained in fish oil. The objective of this study was to determine if dietary PUFA enrichment decreases mucus production or the inflammatory response associated with ovalbumin (OVA)-induced allergic lung inflammation. Mice (n = 10/group) were fed control, 20% fish oil, or 20% corn oil enriched diets for a total of 12 weeks. At 8 and 10 weeks, mice were given an intraperitoneal injection of saline (10 control-fed mice) or OVA (30 remaining mice). Once at 10 weeks and on 3 consecutive days during week 12, mice were challenged by nebulizing with saline or OVA. Mice were euthanized 24 hours after the last challenge and blood was collected for plasma FA analysis. Bronchoalveolar lavage (BAL) fluid was collected to determine cell composition and Th2-type cytokine (IL-4, IL-13) concentrations. Periodic acid-Schiff (PAS) + mucus-producing cells and CD45+ inflammatory cell infiltrates in lung tissue were quantified using morphometric analysis. Relative abundance of mRNA for mucin (Muc4, Muc5ac, and Muc5b) and Th2-type cytokine (IL-4, IL-5, and IL-13) genes were compared with ß-actin by qPCR. Supplementation with either corn oil or fish oil effectively altered plasma FA profiles towards more (n-6) FA or (n-3) FA, respectively (*P* < 0.0001). Sensitization and challenge with OVA increased the proportion of neutrophils, lymphocytes, and eosinophils, and decreased the proportion of macrophages and concentrations of IL-13 in BAL fluid; increased the percentage of PAS+ mucus-producing cells and CD45+ inflammatory cell infiltrates in lung tissue; and increased gene expression of mucins (Muc4, Muc5ac, and Muc5b) and Th2-type cytokines (IL-5 and IL-13) in lung tissue of control-fed mice. Dietary PUFA reversed the increase in PAS+ mucus-producing cells (*P* = 0.003). In addition, dietary enrichment with fish oil attenuated the percentage of CD45+ inflammatory cell infiltrates in lung tissue, and increased Muc4 and Muc 5b gene expression compared with OVA-sensitized and challenged control mice. In conclusion, dietary enrichment with either (n-3) or (n-6) PUFA decreased mucus production in lung tissues of OVA-sensitized and challenged mice. More specifically, enrichment with dietary (n-3) PUFA decreased CD45+ inflammatory cell infiltrates, thus inducing potentially beneficial changes in lung tissue of OVA-sensitized and challenged mice.

## Introduction

The incidence of asthma tends to increase as populations develop more sanitary and affluent Western lifestyles [1]. As communities become more urbanized, patterns of food consumption change [2]. The increased prevalence of asthma over the past several decades could, in part, reflect increased dietary intake of (n-6) polyunsaturated fatty acids (PUFA) and decreased dietary intake of (n-3) PUFA, such as eicosapentaenoic acid (EPA) and docosahexaenoic acid (DHA) contained in fish oil [2, 3]. Based on the anti-inflammatory properties associated with (n-3) PUFA, e.g., EPA and DHA metabolites, and generally pro-inflammatory properties associated with (n-6) PUFA, e.g., linoleic acid (LA) and arachidonic acid (AA) metabolites, some have hypothesized that dietary PUFA content could predisposes certain individuals to chronic inflammatory disorders, such as rheumatoid arthritis, inflammatory bowel disease, and asthma [2].

The hallmark features of asthma include eosinophilic inflammation, mucus hypersecretion, bronchoconstriction, and airway remodeling. Epidemiological and clinical studies using (n-3) FA in asthma and allergic disease were recently reviewed and most indicated protective effects [4]. Although β2-agonists, inhaled corticosteroids, and leukotriene receptor antagonists, along with environmental manipulation, remain the cornerstones of asthma treatment, dietary intervention is an attractive option. A meta-analysis of 26 studies (both randomized placebo-controlled and others) concluded that no definitive conclusion can yet be drawn regarding the efficacy of (n-3) FA supplementation as a treatment for asthma in children and adults (reviewed in [5]). However, many confounding variables existed among studies, including differences in fish oil dose or amounts of EPA and DHA administered. These differences could explain variable outcomes (reviewed in [2, 4, 6]).

As reviewed in detail previously [7], linoleic acid, found in corn, safflower, and soybean oils, and α-linolenic acid (ALA), found in linseed, canola, and soybean oils, are both essential FA and the respective precursors of the (n-6) and (n-3) series of FA. Further metabolism of LA, including elongation and desaturation, produces AA, whereas further metabolism of ALA produces EPA and DHA; the latter are also obtained directly by consumption of fish or fish oil. EPA and DHA typically comprise approximately 30% of the FA present in fish oil [8].

Increasing the amount of dietary (n-3) PUFA relative to (n-6) PUFA results in (n-3) PUFA being preferentially incorporated into cell membrane phospholipids compared with (n-6) PUFA [9, 10]. When cells are activated by a chemical or physical insult, FA are mobilized from cell membrane phospholipids and metabolized into eicosanoids, which are involved in modulating the intensity and duration of inflammatory responses [5]. As previously described in detail [7], further metabolism of AA by cyclooxygenase leads to production of pro-inflammatory eicosanoids of the 2-series, e.g., prostaglandin (PG) E_2_, whereas further metabolism of AA by 5-lipoxygenase yields the 4-series eicosanoids, e.g., leukotriene (LT) B_4_ and the cysteinyl leukotrienes (LTC_4_, LTD_4_, LTE_4_) [5, 10–12]. Alternatively, further metabolism of EPA produces less inflammatory eicosanoids of the 3- and 5-series (e.g., PGE_3_; LTB_5_ and LTE_5_) [5, 10–13]. The functional significance is that mediators formed from EPA are believed to be less inflammatory than those formed from AA [5]. For example, LTB_5_ is 10- to 100-fold less potent as a neutrophil chemoattractant than LTB_4_ [11, 12]. The type of eicosanoids produced, and subsequent communication between immune cells, can potentially be modulated through dietary supplementation of essential PUFA [5, 10–13].

Leukotrienes are potent mediators of inflammatory responses. An exaggerated production of AA-derived leukotrienes has been implicated as the chemical trigger for inflammation in patients with asthma (reviewed by Wong [6]). The AA-derived eicosanoids, e.g., the cysteinyl LTs, are produced by cells that mediate pulmonary inflammation in asthma (e.g., mast cells) [5]. Leukotrienes bind to specific target receptors and directly induce bronchoconstriction and mucus production in the airways. Individuals with asthma produce significantly greater quantities of leukotrienes than nonasthmatic individuals [14]. PGE_2_ is also involved in regulating the development of Th2 lymphocytes that promotes allergic inflammation and formation of IgE by B lymphocytes (reviewed in [5]). Consuming fish oil, results in partial replacement of AA in cell membranes by EPA and DHA, changing the types of eicosanoids produced. Hence, (n-3) FA could have a role in asthma prevention or therapy.

EPA and DHA are also transformed into lipid mediators called resolvins, protectins and maresins with antiinflammary, inflammation-resolving, and immunomodulatory actions (reviewed in [4, 8]. Recently, maresin-1 was shown to target regulatory T cells and type 2 innate lymphoid cells to limit allergic lung inflammation [15].

Regulation of mucus production is necessary for maintenance of mucociliary function, an important component of the innate immune system in the lungs [16–18]. Mucus traps foreign particles and pathogens and dissolve noxious gases, thus facilitating their clearance from the lungs by means of mucociliary transport or cough [19]. Respiratory tract mucus requires the correct combination of viscosity and elasticity for optimal efficiency of mucociliary clearance. Chronic hypersecretion of airway mucus contributes to the pathophysiology of asthma [17]. As described in detail previously, overproduction of mucus, altered viscoelasticity, and buildup of mucus in airway lumens are classic features of chronic airway diseases [16]. Mucins impart to mucus its viscoelastic properties [16]. Muc5ac and Muc5b (which has different glycosylated variants) are key determinants of the physical properties of mucus [19]. The relative amounts of Muc5ac and the glycosylated variants of Muc5b are altered in airway disease, thus compromising transport properties of mucus gel [19].

The goal of our study was to eliminate uncontrolled variables plaguing human studies by utilizing a mouse mode of asthma (OVA-induced lung inflammation) in a controlled laboratory setting. Mice provide an animal model whereby dietary intake can be controlled, there is genetic homogeneity, and tissue sampling is possible. Mice were sensitized (intraperitoneally) and then challenged (intranasally) with OVA to recreate pathologic changes that mimic many of the features of human asthma (reviewed in [20, 21]). We then examined the influence of dietary (n-3) FA from fish oil versus (n-6) FA from corn oil on mucus production and inflammatory responses in airways of mice after OVA-induced allergic lung inflammation. We assessed cellularity and cytokine concentrations in bronchoalveolar lavage (BAL) fluid, the abundance of periodic acid-Schiff (PAS) + mucus-producing cells and CD45+ inflammatory cells in lung tissue, and gene expression in lung tissue of selected mucins and Th2 cytokines. We hypothesized that increased dietary intake of (n-3) PUFA would attenuate features of asthma in mice with OVA-induced allergic lung inflammation.

## Methods

### Mice and diets

Forty female, 6-week-old, BALB/c mice (Jackson Laboratory, Bar Harbor, ME) were randomly distributed into 4 groups (n = 10 each) and housed under conventional conditions. All aspects of this study were approved by the Institutional Animal Care and Use Committee of Oregon State University (ACUP Number: 3397). The first two groups of mice were fed control food (Rodent Diet 8604; Harlan Teklad Premier Laboratory Diets, Madison, WI) for 8 weeks and then sensitized and challenged with saline (**Negative Control**) or OVA (**Positive Control**). The two experimental diet groups received additional PUFA in the form of 20% corn oil (**Corn Oil, High (n-6) FA**; 960246 high corn oil (20%) diet, pelleted; MP Biomedicals) or 20% menhaden fish oil (**Fish Oil, High (n-3) FA;** 960195 high menhaden oil (20%) diet, pelleted; MP Biomedicals, Irvine, CA) for 8 weeks and were then sensitized and challenged with OVA. Mice were provided with food and tap water *ad libitum*. Food intake and body weights were recorded weekly. The caloric density of all foods, average nutrient composition, food ingredients, and vitamin and mineral contents are summarized in Table 1.

**Table 1:** Average nutrient composition (dry matter basis) of diets fed to mice for 12 weeks.

Nutritional Analysis	Control^1^	Corn Oil High (n-6) FA^2^	Fish Oil High (n-3) FA^3^
**Gross energy, kcal/g**	3.93	4.64	4.64
**Nitrogen-free extract, %**	53.6	55.7	55.0
**Crude fat, %**	5.0	20.8	21.5
**Crude protein, %**	28.1	18.8	18.8
**Ash, %**	9.0	3.7	3.2
**Crude fiber, %**	4.2	0.9	1.4

^1^Food ingredients included dehulled soybean meal, wheat middlings, flaked corn, ground corn, fish meal, cane molasses, ground wheat, dried whey, soybean oil, brewers dried yeast, and vitamins and minerals. Minerals included 1.36% calcium as dicalcium phosphate and calcium carbonate, 1.01% phosphorus, 0.29% sodium as iodized salt, 0.49% chlorine, 1.04% potassium as chromium potassium sulfate, 0.28% magnesium as magnesium oxide, 352.14 mg/kg iron as ferrous sulfate, 105.39 mg/kg manganese as manganous oxide, 82.87 mg/kg zinc as zinc oxide, 24.42 mg/kg copper as copper sulfate, 2.46 mg/kg iodine as calcium iodate, 0.71 mg/kg cobalt as cobalt carbonate, and 0.33 mg/kg selenium. Vitamins included 12.90 IU/g vitamin A as vitamin A acetate, 2.40 IU/g vitamin D_3_, 90.18 IU/kg vitamin E, 2.53 mg/g choline as choline chloride, 63.42 mg/kg niacin, 21.03 mg/kg pantothenic acid as calcium pantothenate, 12.95 mg/kg vitamin B_6_ as pyridoxine hydrochloride, 7.85 mg/kg vitamin B_2_, 27.95 mg/kg vitamin B_1_ as thiamine mononitrate, 4.11 mg/kg vitamin K_3_ as menadione sodium bisulfate complex, 2.72 mg/kg folic acid, 0.39 mg/kg biotin, and 51.20 mcg/kg vitamin B_12_.

^2^Food ingredients (by weight) included 35.58% sucrose, 20.00% casein purified high nitrogen, 20.00% corn oil, 15.00% corn starch, 5.00% alphacel, non-nutritive bulk, 4.00% mineral mix, 0.30% dl-methionine, and 0.12% dl-α-tocopherol powder (250 IU/g). The mineral mix contained (g/kg mineral mix): 500 g calcium phosphate dibasic, 220 g potassium citrate monohydrate, 74 g sodium chloride, 52 g potassium sulfate, 24 g magnesium oxide, 6 g ferric citrate, 3.5 g manganese carbonate, 1.6 g zinc carbonate, 0.55 g chromium potassium sulfate, 0.3 g cupric carbonate, 0.01 g potassium iodate, and 0.01 g sodium selenite all in 118 g sucrose. Vitamins were added at 1.2 x normal amount of MP Biomedicals vitamin diet fortification mixture.

^3^Food ingredients (by weight) included 34.58% sucrose, 20.00% casein purified high nitrogen, 20.00% menhaden oil, 15.00% corn starch, 5.00% alphacel, non-nutritive bulk, 4.00% mineral mix, 1.00% corn oil, 0.30% dl-methionine, and 0.12% dl-α-tocopherol powder (250 IU/g). The mineral mix contained (g/kg mineral mix): 500 g calcium phosphate dibasic, 220 g potassium citrate monohydrate, 74 g sodium chloride, 52 g potassium sulfate, 24 g magnesium oxide, 6 g ferric citrate, 3.5 g manganese carbonate, 1.6 g zinc carbonate, 0.55 g chromium potassium sulfate, 0.3 g cupric carbonate, 0.01 g potassium iodate, and 0.01 g sodium selenite all in 118 g sucrose. Vitamins were added at 1.2 x normal amount of MP Biomedicals vitamin diet fortification mixture.

### Allergen Sensitization and Challenge Protocol

**Fig 1:**
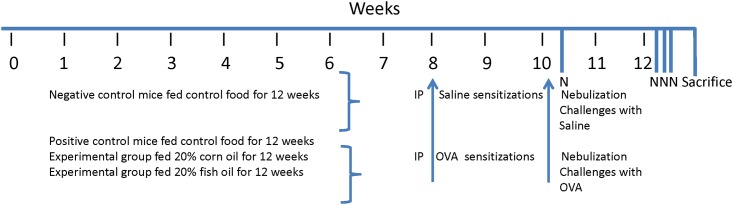
Time line for the experimental protocol. Mice continued to eat their respective foods throughout the allergen sensitization and challenge period. They were sensitized with saline or OVA at 8 and 10 weeks, and then challenged by nebulization with saline or OVA once at 10 weeks and on three consecutive days during week 12. Mice were sacrificed 24 h after the final nebulization.

The time line for the experimental protocol is illustrated in Fig 1. Mice continued to eat their respective foods throughout the allergen sensitization and challenge period, described as follows [21]. At 8 and 10 weeks, all mice, except for Negative Control mice, were given intraperitoneal injections of OVA (100 μg; 0.2 mL) complexed with aluminum potassium sulfate (alum; both from Sigma Chemical Company, St. Louis, MO). Negative Control mice were injected intraperitoneally with an equivalent volume of saline. Once at 10 weeks and on 3 consecutive days during week 12, mice were challenged by nebulization with saline (Negative Control) or OVA (remaining groups) for 60 min. For nebulization, mice were placed in a 40.6 cm x 20.3 cm x 25.4 cm plexiglass nebulization chamber (Nebulair Veterinary Portable Ultrasonic Nebulizer, Model KUN-88; DVM Pharmaceuticals, Miami, FL) and nebulized with 0.9% saline (group 1) or 1% OVA in 0.9% saline (groups 2, 3, and 4). At 24 h after the final nebulization, all mice were anesthetized with 0.25 mL of a mixture of ketamine (7.6 mg/mL; Ketaset^®^, Fort Dodge Animal Health, Fort Dodge, IA) and medetomidine (0.1 mg/mL; Domitor^®^, Pfizer Animal Health, New York, NY) and euthanized by cervical dislocation following American Veterinary Medical Association guidelines, and as approved by the Institutional Animal Care and Use Committee of Oregon State University (ACUP Number: 3397).

### Plasma fatty acid analyses

Whole blood was collected by exsanguination into EDTA. Plasma was separated from blood and stored at -20°C for later plasma FA analyses. Lipids were extracted from food and plasma as described by Folch et al. [22] using chloroform:methanol (2:1, vol/vol). Briefly, after an overnight incubation at 4°C, a sodium chloride solution (0.88%) was added and mixed. The phases were separated by centrifugation and the lower chloroform layer was collected for FA analysis. Two mL of lipid extract were dried under nitrogen, resolubilized in 3 mL boron trifluoride-methanol (10% wt/wt), and esterified by heating to 95°C. The FA methyl esters were determined as previously described [23]. Fatty acid analysis was performed with an HP 6890 gas chromatograph equipped with an autosampler, flame ionization detector, and SP-2330 fused silica capillary column (30 mm × 0.25 mm i.d). Fatty acid methyl esters were identified by comparing retention times of authentic standards (Nuchek Prep, Elysian, MN). Peak areas and percentages were calculated using Hewlett Packard ChemStation software (Agilent Technologies Inc, Wilmington, DE). The FA data are reported as weight % of total FA (g/100g FA).

### Bronchoalveolar lavage fluid cytology

The trachea was exposed and cannulated with an intravenous catheter (Surflo i.v. catheter, 18 g x 6.4 cm; Butler Animal Health Supply, Visalia, CA). The lungs were manually lavaged with 0.5 mL phosphate buffered saline (PBS) three times. For each lavage, the fluid was washed in and out twice before collection. The recovered BAL fluid was collected and 200 uL removed for differential cell counts of macrophages, neutrophils, eosinophils, and lymphocytes from cytospin preparations of cells stained with Wright Giemsa. Two hundred cells were counted on each slide. Results are expressed as percentages of total cell count.

### Bronchoalveolar lavage fluid cytokines (IL-4 and IL-13) assays

Concentrations of IL-4 (≥4 pg/mL) and IL-13 (≥30 pg/mL) in the BAL fluid were determined by ELISA (eBioscience, San Diego, CA) according to manufacturer’s instructions. The mouse IL-4 ELISA (Catalog Number 88–7944) and IL-13 ELISA (Catalog Number 88–7134) assays used undiluted BAL fluid samples. Results are expressed as pg/mL.

### Lung histopathology and immunohistochemistry

The right lung was tied off, excised, snap frozen in liquid nitrogen, and stored at -80°C for later analysis. The trachea and all lobes of the left lung were lavaged with 0.3 mL of 10% neutral buffered formalin, excised, and placed in a formalin jar for histopathological analysis. Lung tissues fixed in 10% neutral buffered formalin were sectioned to obtain an anterior-to-posterior representation of the entire left lung, embedded in paraffin, and cut into stepwise sections (~50 μm separation). Individual sections (5 μm thick) were stained with PAS stain for mucus and anti-CD45 monoclonal antibody for detecting inflammatory cell infiltrates. Briefly, for the PAS staining process for mucus, 0.5% PAS reagent (Thermo Fisher Scientific; Waltham, MA) was warmed in a 37°C oven for 1 h. Slides were deparaffinized and hydrated using distilled water. Slides were then treated with 0.5% periodic acid at room temperature for 5 min and rinsed in 2 changes of distilled water. Slides were placed in a Coplin jar with 50 mL of Schiff’s reagent and irradiated at full power for 10 s in a microwave oven. Slides were allowed to sit in the solution for 5 min followed by a running water wash for 10 min, and a final rinse in distilled water. Slides were counterstained for 30 s in Harris hematoxylin (Thermo Fisher Scientific), washed in running water for 5 min, and then rinsed in 2 changes of distilled water. Slides were subsequently dehydrated with two changes each of 95% ethanol, 100% ethanol, and xylene, and then mounted with a cover slip applied with synthetic mounting medium.

For the anti-CD45 monoclonal antibody staining process, sectioned tissues were collected on charged slides and baked at 60°C for 1 h. Slides were rehydrated through 2 changes of xylene, 2 changes of 100% ethanol, 1 change of 80% ethanol, and water. High temperature antigen retrieval was performed in a microwave pressure cooker (Viking Tender Cooker; Nordicware, Minneapolis, MN) using a target retrieval solution (Dako; Carpenteria, CA) for 10 min after pressure was reached. The pressure cooker was slowly vented and the container containing the slides was allowed to sit for 20 min at room temperature. Slides were placed on an autostainer (Dako) and washed in Tris buffered saline with Tween (TBST) followed by 3% hydrogen peroxide in TBST for 5 min, and then a serum-free protein block (Dako) was added for 10 min, followed by rinsing with TBST. Endogenous biotin was blocked using a blocking kit (Vector Laboratories; Burlingame, CA). An avidin solution was applied for 15 min, then slides were rinsed with TBST; a biotin solution was added for an additional 15 min; then slides were again rinsed in TBST. The primary antibody, biotinylated-rat-anti-CD45 (Genetex GTX19592; San Antonio, TX) was diluted 1:200 in diluent (Dako) and applied for 30 min at room temperature. Normal rat serum (Vector Laboratories) was diluted at the same ratio as the primary antibody and served as the negative control. After washing slides in TBST, a prediluted strepavidin-horseradish peroxidase (SAv-HRP; BD Pharmingen; San Diego, CA) was applied for 30 min at room temperature and then washed off with TBST. The chromagen Nova Red (Vector Laboratories) was applied for 5 min and washed off using distilled water, followed by hematoxylin (Dako) application for 5 min, rinsing in distilled water, rinsing in TBST to blue the counter stain, rinsing again with distilled water, and then dehydrating with two changes each of 95% ethanol, 100% ethanol, and xylene. Finished slides were mounted with a cover slip.

### Lung histopathology and morphometric analysis

Non-overlapping digital images from the entire section of lung were captured with a Nikon Eclipse 50i compound light microscope (Nikon Instruments, Melville, NY) equipped with a Canon EOS Digital Rebel XT color camera (Canon U.S.A., Lake Success, New York) interfaced to a personal computer running Windows XP. The camera was linked to the computer using camera control software, DSLR Remote Pro (Breeze Systems, Bagshot, UK), and images were saved with Adobe Photoshop CS2 image capture software version 9.0.2. Final magnification of images analyzed was 25X. Computerized image analysis (Image-Pro^®^ Plus Version 5.1; MediaCybernetics, Bethesda, MD) was used for quantitative evaluation of PAS+ mucus-producing cells or CD45+ inflammatory cell aggregates in the captured images, using methods similar to those reported in Schwindt et al. [24]. Briefly, a color filtering algorithm was developed to select colors represented by either PAS+ mucus-producing cells or CD45+ cells and cell aggregates. The color-filter algorithms were constructed from a composite image of PAS+ mucus-producing cells or CD45+ aggregates cut from a subset of all images. Cells from each treatment were equally represented in their respective composite image. Magenta color (PAS stain) or red-brown color (Nova Red chromagen in CD45 preparations) was selected on composite images, and algorithms were saved and applied to all respective images. Background or non-specific color selection was not observed. The area occupied by the respective stain per total image area in pixels, was exported to an Excel^®^ spreadsheet and the median percent of pigmented tissue area of 3 to 6 counted slides per mouse are reported.

### Quantitation of selected mRNAs specific for mucin (Muc4, Muc5ac, and Muc5b) and Th2 cytokine (IL-4, IL-5, and Il-13) gene expression

The right lung of each mouse, which had previously been tied off, excised, snap frozen in liquid nitrogen, and stored at -80°C, was used to measure gene expression of selected mRNA. An approximately 20 mg piece of frozen lung was placed into 600 μl of RLT buffer (Qiagen Sciences; Germantown, MD), and immediately processed using a Tissue Tearor^™^ (Biospec Products, Inc., Model 398, Dremel; Racine, WI). Total RNA was isolated (RNeasy^®^ Mini Kit, Qiagen Sciences) using manufacturer’s instructions. RNA was quantitated with a ND-1000 NanoDrop Spectrophotometer (Thermo Fisher Scientific; Waltham, MA). Preparations were stored at -80°C. Subsequent aliquots of 500 ng of total RNA were processed to cDNA using the SABiosciences (Frederick, MD) C01 cDNa kit. To assess mucin (Muc4, Muc5ac, and Muc5b) and cytokine (IL-4, IL-5, IL-13) gene expression, relative abundances of mRNA for these selected genes were measured and compared with β-actin by qPCR using the 2x SybrGreen^™^ assay mix specific for Applied Biosystems equipment (Foster City, CA) supplied by SABiosciences. The proprietary qPCR primers for these genes were also supplied by SABiosciences. Data were analyzed as ΔΔCt-values [25]. Results are expressed as fold changes compared with Negative Control mice.

### Statistical analyses

Statistical analyses were performed using SAS version 9.3 (SAS Institute, Cary, NC). Lung tissue PAS staining and BAL fluid IL-4 concentrations both had 3 outliers (one in each OVA sensitized and challenged) group; thus PAS staining above 0.31% were set at 0.31% and IL-4 concentrations above 50 pg/mL were set at 50 pg/mL. The outliers were identified using box plots and differed more than 1.5 interquartile ranges from the 75 percentile values. After testing for normal distribution and equal variance, endpoint data were analyzed using one-way ANOVA with PROC GLM and treatment (Negative Control, Positive Control, Corn Oil, and Fish Oil) as fixed effect. Repeated measures in time (body weight and feed intake) were analyzed using repeated-measures-in-time analysis with PROC MIXED; the fixed effects in the statistical model were treatment, week, and their interactions. The variance-covariance matrix of repeated measurements within animal was modeled using a first-order autoregressive homogeneous variance covariance matrix.

To evaluate OVA sensitization, data of Positive Control mice were compared with those of Negative Control mice. To evaluate the effect of PUFA supplementation, data of Corn Oil and Fish Oil mice were compared with those of Positive Control mice. To evaluate the effect of PUFA type, data of Fish Oil mice were compared with those of Corn Oil mice. All statistical tests were two-sided and significance was set at *P* ≤ 0.05.

## Results

### Dietary and plasma fatty acid profiles

The FA composition of the diets is shown in Table 2. Total (n-3) FA concentration was higher in the fish oil-enriched diet compared with control or corn oil-enriched diets. The fish oil-enriched diet also contained the most saturated fatty acids (SFA) and the least total PUFA. The control and corn oil-enriched foods had higher total (n-6) FA concentration compared with fish oil-enriched food. The ratio of (n-6) to (n-3) FA was 51.5:1, 8.1:1, and 0.2:1 for the corn oil-enriched, control, and fish oil-enriched diets, respectively.

**Table 2:** Composition of selected FA in diets fed to mice for 12 weeks.

Fatty Acid (g/100 g FA)	Control	Corn Oil High (n-6) FA	Fish Oil High (n-3) FA
**14:0**	0.6	0.4	7.4
**16:0**	15.5	12.7	17.3
**18:0**	3.5	1.8	3.0
**Total SFA**^1^	20.1	14.9	28.4
**18:1 (n-9)**	24.7	24.2	5.9
**Total MUFA**^2^	26.4	24.2	19.8
**18:2 (n-6)**	46.9	52.5	4.6
**18:3 (n-3)**	4.9	1.0	1.5
**20:4 (n-6)**	<0.01	<0.01	1.1
**20:5 (n-3)**	0.5	<0.01	11.0
**22:6 (n-3)**	0.4	<0.01	10.8
**Total PUFA**^3^	52.7	53.5	32.3
**Total (n-6) FA**^4^	46.9	52.5	6.1
**Total (n-3) FA**^5^	5.8	1.0	25.4
**(n-6):(n-3)**	8.1:1	51.5:1	0.2:1

^1^Total saturated fatty acids (SFA): 14:0 + 15:0 + 16:0 + 17:0 + 18:0 + 20:0 + 22:0 + 24:0.

^2^Total monounsaturated fatty acids (MUFA): 14:1 + 15:1 + 16:1n-7 + 17:1 + 18:1n-9*c* + 18:1n-7 + 18:1n-9*t* + 20:1n-9 + 22:1n-9 + 24:1.

^3^Total polyunsaturated fatty acids (PUFA) = 18:2n-6 + 18:3n-6 + 18:3n-3 + 18:4n-3 + 20:2n-6 + 20:3n-6 + 20:3n-3 + 20:4n-6 + 20:4n-3 + 20:5n-3 + 21:5n-3 + 22:2n-6 + 22:4n-6 + 22:5n-6 + 22:5n-3 + 22:6n-3.

^4^Total n-6 FA: 18:2n-6 + 18:3n-6 + 20:2n-6 + 20:3n-6 + 20:4n-6 + 22:2n-6 + 22:4n-6 + 22:5n-6.

^5^Total n-3 FA: 18:3n-3 + 18:4n-3 + 20:3n-3 + 20:4n-3 + 20:5n-3 + 21:5n-3 + 22:5n-3 + 22:6n-3.

Plasma FA profiles are shown in Table 3. Sensitization of mice with OVA did not significantly alter plasma FA profiles. However, dietary inclusion of PUFA in OVA-sensitized and challenged mice decreased the proportion of total MUFA in plasma FA compared with Positive Controls (*P*<0.0001), and changed total PUFA, including (n-6) and (n-3) FA, compared with Positive Controls (all *P*≤0.0002). Consistent with their dietary FA profile (Table 2), OVA-sensitized and challenged mice fed corn oil had a lower proportion of SFA, including 14:0, 16:0, and 18:0, and higher proportion of 18:1 (*P*<0.001) and total PUFA (*P*<0.0001) compared with OVA-sensitized and challenged mice fed fish oil. As expected, OVA-sensitized and challenged mice consuming a high EPA and DHA diet, had a higher proportion of EPA, DHA, and total (n-3) FA in plasma compared with OVA-sensitized and challenged mice consuming a high (n-6) FA diet. The proportion of LA, AA, and total (n-6) FA in plasma FA were lower in OVA-sensitized and challenged mice consuming a high EPA and DHA diet, resulting in a large difference in the ratio of (n-6) to (n-3) FA (all *P*<0.0001) compared with OVA-sensitized and challenged mice consuming a high (n-6) FA diet.

**Table 3:** Plasma concentrations (g/100 g FA) of selected FA in mice after dietary intervention for 12 weeks^1^.

	Diets				P-Values		
Fatty Acid (g/100 g FA)	Negative Control	Positive Control	Corn Oil High (n-6)	Fish Oil High (n-3)	Induction^2^	PUFA^3^	PUFA Type^4^
	N = 3	N = 3	N = 7	N = 7			
**14:0**	0.6 ± 0.6	nd	nd	1.3 ± 0.4	0.54	0.36	0.04
**16:0**	22.3 ± 0.7^a^	21.1 ± 0.7^a^	17.1 ± 0.5^b^	21.9 ± 0.5^a^	0.27	0.06	<0.0001
**18:0**	9.6 ± 0.7^b^	9.2 ± 0.7^b^	10.9 ± 0.4^a^	12.3 ± 0.4^a^	0.71	0.005	0.04
**Total SFA**^5^	32.4 ± 1.1^b^	30.3 ± 1.1^bc^	28.0 ± 0.7^c^	35.5 ± 0.7^a^	0.20	0.25	<0.0001
**18:1 (n-9)**	18.0 ± 1.0^a^	19.0 ± 1.0^a^	13.8 ± 0.6^b^	9.9 ± 0.6^c^	0.45	<0.0001	0.0006
**Total MUFA**^6^	20.4 ± 1.1^a^	21.7 ± 1.1^a^	14.1 ± 0.7^b^	14.3 ± 0.7^b^	0.43	<0.0001	0.82
**18:2 (n-6)**	28.0 ± 1.0^b^	27.7 ± 1.0^b^	34.3 ± 0.6^a^	8.8 ± 0.6^c^	0.79	<0.0001	<0.0001
**18:3 (n-3)**	0.4 ± 0.1	nd	nd	nd	0.06	1.00	1.00
**20:4 (n-6)**	15.0 ± 1.2^b^	14.6 ± 1.2^b^	19.8 ± 0.8^a^	12.0 ± 0.8^b^	0.84	0.36	<0.0001
**20:5 (n-3)**	nd	nd	nd	14.7 ± 0.2	1.00	<0.0001	<0.0001
**22:6 (n-3)**	3.4 ± 0.5^bc^	4.9 ± 0.5^b^	2.5 ± 0.3^c^	14.4 ± 0.3^a^	0.07	<0.0001	<0.0001
**Total PUFA**^7^	47.2 ± 1.1^c^	48.0 ± 1.1^bc^	57.9 ± 0.7^a^	50.1 ± 0.7^b^	0.63	0.0002	<0.0001
**Total (n-6) FA**^8^	43.4 ± 0.9^b^	43.1 ± 0.9^b^	55.4 ± 0.6^a^	20.8 ± 0.6^c^	0.83	0.0002	<0.0001
**Total (n-3) FA**^9^	3.8 ± 0.7^bc^	4.9 ± 0.7^b^	2.5 ± 0.5^c^	29.4 ± 0.5^a^	0.33	<0.0001	<0.0001
**(n-6):(n-3)**	15.2 ± 2.8^b^	8.9 ± 0.8^b^	23.0 ± 1.8^a^	0.7 ± 1.8^c^	0.14	0.36	<0.0001

^1^Values are LSM ± SEM; nd is not detected.

^abc^Mean values within a row with different letters are significantly different at *P* < 0.05.

^2^Induction: Positive Control vs. Negative Control

^3^PUFA: Corn and fish oil vs. Positive Control

^4^PUFA Type: Fish oil vs. corn oil

^5^Total saturated fatty acids (SFA): 14:0 + 15:0 + 16:0 + 17:0 + 18:0 + 20:0 + 22:0 + 24:0.

^6^Total monounsaturated fatty acids (MUFA): 14:1 + 15:1 + 16:1n-7 + 17:1 + 18:1n-9*c* + 18:1n-7 + 18:1n-9*t* + 20:1n-9 + 22:1n-9 + 24:1.

^7^Total polyunsaturated fatty acids (PUFA): 18:2n-6 + 18:3n-6 + 18:3n-3 + 18:4n-3 + 20:2n-6 + 20:3n-6 + 20:3n-3 + 20:4n-6 + 20:4n-3 + 20:5n-3 + 21:5n-3 + 22:2n-6 + 22:4n-6 + 22:5n-6 + 22:5n-3 + 22:6n-3.

^8^Total n-6 FA: 18:2n-6 + 18:3n-6 + 20:2n-6 + 20:3n-6 + 20:4n-6 + 22:2n-6 + 22:4n-6 + 22:5n-6.

^9^Total n-3 FA: 18:3n-3 + 18:4n-3 + 20:3n-3 + 20:4n-3 + 20:5n-3 + 21:5n-3 + 22:5n-3 + 22:6n-3.

### Body weights and food consumption

All mice readily consumed their respective control or PUFA-enriched diets. Sensitization of control mice with OVA did not affect final body weights compared with Negative Control mice (23.5 ± 0.6 g versus 23.9 ± 0.6 g; *P* = 0.61); however, PUFA supplementation of OVA-sensitized and challenged mice was associated with lower body weights throughout the feeding period compared with Positive Control mice (19.5 ± 0.2 g versus 21.1 ± 0.3 g; *P* = 0.0009). The body weights of OVA-sensitized and challenged mice receiving corn oil versus fish oil were not significantly different throughout the feeding period (19.9 ± 0.3 g versus 19.0 ± 0.3 g; *P* = 0.08). Treatment group differences in body weights were consistent with group differences in weekly food intake, as PUFA supplemented OVA-sensitized and challenged mice had decreased weekly food intake compared with mice consuming control diet (21.7 ± 0.6 g versus 27.2 ± 0.7 g; *P* < 0.0001), with no significant group differences in weekly food intake between OVA-sensitized and challenged mice receiving corn oil versus fish oil (21.1 ± 0.8 g versus 22.3 ± 0.8 g; *P* = 0.30).

### Bronchoalveolar lavage fluid cytology

Cytology proportions in BAL fluid are shown in Table 4. Sensitization with OVA altered the BAL cytology profile in control mice, increasing the proportion of eosinophils and lymphocytes and decreasing the proportion of macrophages (*P* ≤0.0002). Inclusion of PUFA did not result in consistent changes in BAL cytology of OVA-sensitized and challenged mice. In OVA-sensitized and challenged mice consuming fish oil, the proportion of neutrophils was lower (*P* = 0.004) and the proportion of eosinophils was higher (*P* = 0.003) compared with OVA-sensitized and challenged mice consuming corn oil.

**Table 4:** BAL fluid cytology proportions for mice fed (n-3) or (n-6) PUFA enriched foods for 8 weeks and then sensitized and challenged with OVA for an additional 4 weeks compared with mice fed control food that were sensitized and challenged with saline (Negative Control) or OVA (Positive Control)^1^.

	Diets				P-Values		
Cell Type (% of Total)	Negative Control	Positive Control	Corn Oil High (n-6)	Fish Oil High (n-3)	Induction^2^	PUFA^3^	PUFA Type^4^
	N = 7	N = 10	N = 10	N = 9			
**Neutrophils**	11.8 ± 4.5^b^	22.1 ± 3.8^b^	40.5 ± 3.8^a^	23.3 ± 4.0^b^	0.09	0.05	0.004
**Macrophages**	81.0 ± 4.4^a^	35.2 ± 3.7^b^	25.2 ± 3.7^b^	29.8 ±3.9^b^	<0.0001	0.11	0.40
**Lymphocytes**	7.8 ± 1.9^b^	18.1 ± 1.6^a^	18.4 ± 1.6^a^	15.7 ± 1.7^a^	0.0002	0.57	0.24
**Eosinophils**	nd^c^	23.7 ± 3.2^ab^	15.9 ± 3.2^b^	31.1 ± 3.4^a^	<0.0001	0.96	0.003

^1^Values are LSM ± SEM.

^abc^Mean values within a row with different letters are significantly different at *P* < 0.05.

^2^Induction: Positive Control vs. Negative Control

^3^PUFA: Corn and fish oil vs. Positive Control

^4^PUFA Type: Fish oil vs. corn oil

### Bronchoalveolar lavage fluid IL-4 and IL-13 concentrations

IL-4 and IL-13 cytokine concentrations in bronchoalveolar lavage fluid are shown in Table 5. Sensitization with OVA decreased IL-13 in BAL fluid of control mice (*P* = 0.04), but was not further altered by type of dietary PUFA. Concentrations of IL-4 in BAL fluid were not significantly different (*P* = 0.29) among treatment groups.

**Table 5:** Cytokine (IL-4 and IL-13) concentrations in BAL fluid of mice fed (n-3) or (n-6) PUFA enriched foods for 8 weeks and then sensitized and challenged with OVA for an additional 4 weeks compared with mice fed control food that were sensitized and challenged with saline (Negative Control) or OVA (Positive Control)^1^.

	Diets				P-Values		
Cytokine (pg/mL)	Negative Control	Positive Control	Corn Oil High (n-6)	Fish Oil High (n-3)	Induction^2^	PUFA^3^	PUFA Type^4^
	N = 10	N = 10	N = 10	N = 9			
**Interleukin 4**	10.8 ± 4.9	15.5 ± 4.9	24.0 ± 4.9	14.6 ± 5.2	0.50	0.54	0.19
**Interleukin 13**	344 ± 43^a^	219 ± 43^b^	295 ± 43^ab^	217 ± 45^b^	0.04	0.48	0.21

^1^Values are LSM ± SEM.

^abc^Mean values within a row with different letters are significantly different at *P* < 0.05.

^2^Induction: Positive Control vs. Negative Control

^3^PUFA: Corn and fish oil vs. Positive Control

^4^PUFA Type: Fish oil vs. corn oil

### Morphometric analysis of PAS+ and CD45+ stained lung tissue

**Fig 2:**
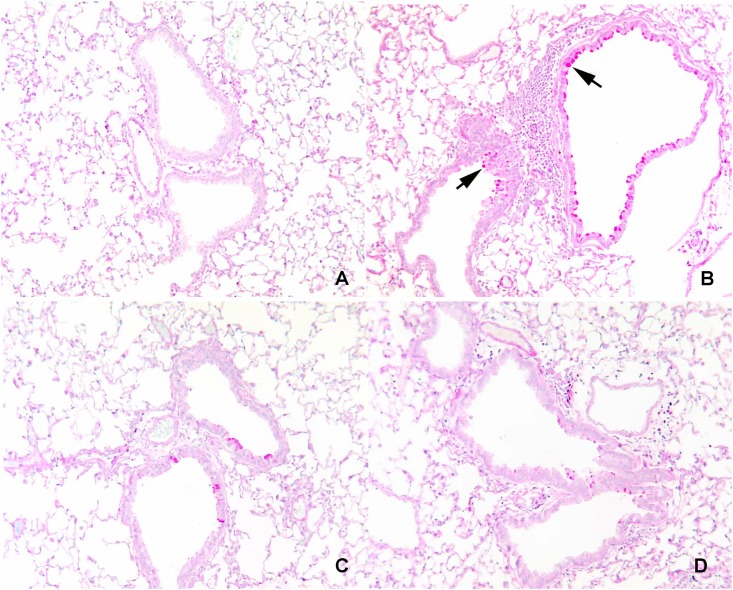
Representative light microscopy images of lung tissue. Lung tissues were stained with periodic acid-Schiff (PAS) stain for mucus after mice were fed their respective foods for a total of 12 weeks. They were sensitized and challenged with saline or OVA starting at 8 weeks. (A) Mucus-positive cells are not evident in most areas of lung of mice fed standard rodent food and sensitized and challenged with saline (Negative Control). (B) Mice fed standard rodent food and sensitized and challenged with OVA (Positive Control) exhibit a marked increase in mucus-producing cells. Arrows indicate PAS+ mucus. (C) Mice fed Corn Oil-enriched food, or (D) Fish Oil-enriched food exhibit less PAS+ staining cells than mice fed standard rodent food and sensitized and challenged with OVA (*P* = 0.003). Original magnification 25X.

**Fig 3:**
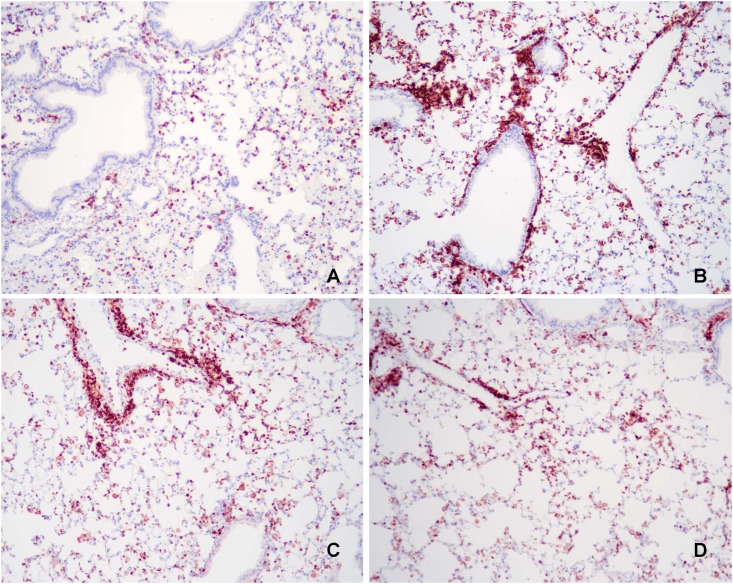
Representative light microscopy images of lung tissue. Lung tissues were stained with rat anti-mouse CD45 after mice were fed their respective foods for 12 weeks. They were sensitized and challenged with saline or OVA starting at 8 weeks. (A) Only circulating leukocytes are seen in control mice fed standard rodent food and sensitized and challenged with saline (Negative Control). (B) Mice fed standard rodent food and sensitized and challenged with OVA (Positive Control) or (C) Corn Oil-enriched food exhibit a marked increase in CD45+ inflammatory cells, particularly peribronchial infiltrates. (D) Overall, there were less CD45+ inflammatory cells in mice fed Fish-Oil enriched food compared with mice fed control food (Positive Control; *P* = 0.04). Original magnification 25X.

PAS+ and CD45+ staining lung tissue are shown in Figs 2 and 3, respectively. Sensitization with OVA increased the percentage of lung staining positive for both PAS and CD45 in control mice (both *P* < 0.0001; Table 6). Inclusion of PUFA in the diet attenuated the percent of PAS+ lung tissue in OVA-sensitized and challenged mice (*P* = 0.003) to levels not significantly different from those of Negative Control mice. Moreover, the percent of lung tissue staining CD45+ was 31% lower in fish-oil supplemented mice than in Positive Control mice (*P* = 0.04).

**Table 6:** Percent of lung tissue staining positive with periodic acid-Schiff (PAS) stain for mucus and rat anti-mouse CD45 stain, after mice were fed (n-3) or (n-6) PUFA enriched foods for 8 weeks and then sensitized and challenged with OVA for an additional 4 weeks compared with mice fed control food that were sensitized and challenged with saline (Negative Control) or OVA (Positive Control)^1^.

	Diets				P-Values		
Positive Stain (% of Total)	Negative Control	Positive Control	Corn Oil High (n-6)	Fish Oil High (n-3)	Induction^2^	PUFA^3^	PUFA Type^4^
	N = 10	N = 10	N = 10	N = 9			
**PAS+**	0.008 ± 0.0028^b^	0.188 ± 0.0028^a^	0.068 ± 0.0028^b^	0.082 ± 0.0030 ^b^	<0.0001	0.003	0.74
**CD45+**	0.67 ± 0.42^c^	4.16 ± 0.42^a^	3.57 ± 0.42^ab^	2.87 ± 0.44^b^	<0.0001	0.08	0.27

^1^Values are LSM ± SEM.

^abc^Mean values within a row with different letters are significantly different at *P* < 0.05.

^2^Induction: Positive Control vs. Negative Control

^3^PUFA: Corn and fish oil vs. Positive Control

^4^PUFA Type: Fish oil vs. corn oil

### Quantitation of selected mRNAs specific for mucin (Muc4, Muc5ac, and Muc5b) and Th2 cytokine (IL-4, IL-5, and Il-13) gene expression in lung tissue

Fold changes in gene expression for selected mRNA in lung tissue are shown in Table 7. Sensitization with OVA induced gene expression of Muc4, Muc5ac, and Muc5b, IL-5, and IL-13 but not IL-4 in control mice (all *P* < 0.004). Of these 6 selected genes, inclusion of dietary PUFA increased only Muc4 gene expression in OVA-sensitized and challenged mice (*P* = 0.03) with greater gene expression observed for mice receiving fish oil versus corn oil (*P* = 0.04). In addition OVA sensitized mice receiving fish oil had greater Muc5b gene expression than mice fed control food (*P* = 0.05).

**Table 7:** Fold changes in selected lung tissue mRNAs specific for mucin (Muc4, Muc5ac, and Muc5b) and Th2 cytokine (IL-4, IL-5, and Il-13) genes, after mice were fed (n-3) or (n-6) PUFA enriched foods for 8 weeks and then sensitized and challenged with OVA for an additional 4 weeks compared with mice fed control food that were sensitized and challenged with saline (Negative Control) or OVA (Positive Control)^1^.

	Diets				P-Values		
Fold Change	Negative Control	Positive Control	Corn Oil High (n-6)	Fish Oil High (n-3)	Induction^2^	PUFA^3^	PUFA Type^4^
	N = 10	N = 10	N = 10	N = 9			
**Mucin 4**	1.00 ± 0.15^c^	2.60 ± 0.37^b^	3.10 ± 0.45^b^	4.87 ± 0.75^a^	<0.0001	0.03	0.04
**Mucin 5ac**	1.00 ± 0.55^b^	27.8 ± 15.2^a^	37.1 ± 20.3^a^	88.9 ± 51.5^a^	<0.0001	0.27	0.26
**Mucin 5b**	1.00 ± 0.40^c^	5.95 ± 2.38^b^	8.43 ± 3.37^ab^	18.6 ± 7.9^a^	0.003	0.13	0.17
**Interleukin 4**	1.00 ± 0.23^b^	1.59 ± 0.36^ab^	1.70 ± 0.39^ab^	2.65 ± 0.64^a^	0.16	0.31	0.19
**Interleukin 5**	1.00 ± 0.12^b^	1.80 ± 0.22^a^	1.42 ± 0.18^a^	1.63 ± 0.21^a^	0.002	0.32	0.37
**Interleukin 13**	1.00 ± 0.22^b^	6.92 ±1.55^a^	9.35 ± 2.10^a^	12.3 ± 2.9^a^	<0.0001	0.12	0.41

^1^Values are LSM ± SEM.

^abc^Mean values within a row with different letters are significantly different at *P* < 0.05.

^2^Induction: Positive Control vs. Negative Control

^3^PUFA: Corn and fish oil vs. Positive Control

^4^PUFA Type: Fish oil vs. corn oil

## Discussion

Airway and pulmonary inflammation were induced in a mouse-asthma model similar to what has been previously described [21, 26]. Using this model, we showed that sensitization and challenge with OVA increased the proportion of eosinophils, neutrophils, and lymphocytes and decreased the proportion of macrophages in BAL fluid; increased the percentage of PAS+ mucus-producing cells and CD45+ inflammatory cell infiltrates in lung tissue; increased gene expression of mucins (Muc4, Muc5ac, and Muc5b); and increased gene expression of Th2-type cytokines (IL-5 and IL-13) in lung tissue of mice fed control diet. We demonstrated that feeding either fish oil or corn oil enriched diets significantly decreased the OVA-induced increase in mucus-producing cells in lung tissues of OVA-sensitized and challenged mice. However, dietary enrichment with fish oil also attenuated the percentage of CD45+ inflammatory cell infiltrates in lung tissue, and increased Muc4 and Muc5b gene expression compared with OVA-sensitized and challenged control mice.

Mice readily consumed their corn oil- and fish oil-enriched diets. Growing mice in all treatment groups gained weight during the 12-week feeding period. Although induction of OVA-airway hypersensitivity did not alter final body weights, PUFA-fed OVA-sensitized and challenged mice did have lower body weights throughout the feeding period consistent with decreased food intake of corn oil and fish oil enriched diets compared with Positive Control mice. However, this study was not designed to conclude that mice of lower body weight are less susceptible to induction of asthma.

Plasma FA profiles of mice reflected PUFA content of diets fed. Total (n-3) FA content of control food was intermediate among the three diets. Plasma concentrations of total (n-3) FA were highest in OVA-sensitized and challenged mice consuming fish oil-enriched food, intermediate in mice consuming control food, and lowest in mice consuming corn oil-enriched food. Although control diet was intermediate in (n-3) FA content, plasma total (n-3) FA concentrations in mice fed control diet were more similar to mice fed corn oil- than fish oil-enriched diets.

We have previously shown that healthy, geriatric Beagle dogs fed foods enriched in (n-3) FA from fish oil for 12 weeks have altered plasma FA profiles [27, 28], similar to what has been reported in humans, horses, and laboratory animals [7, 11, 29, 30]. Incorporation of (n-3) FA into cell membranes is significantly increased within 2 weeks of beginning supplementation and concentrations of EPA reach peak accumulation after 6 weeks of supplementation (reviewed in [6]). Thus, it is likely that membrane phospholipids in inflammatory cells of mice in this study were altered by 8 weeks, at the time of OVA sensitization.

OVA-induced airway inflammation in sensitized mice was associated with mucus hyperproduction as shown by quantitative assessment of PAS+ staining cells using morphometric analysis. Mucus deposition within the airway epithelia was increased. In our study, feeding fish oil or corn oil enriched diets decreased mucus deposition in OVA-sensitized and challenged mice, confirming the findings of a previous report, which also showed that feeding fish oil to male A/J mice reduced mucus production [26]. In addition, although total leukocyte numbers in BAL fluids in our study were not measured, relative proportions of differential cell types in airway BAL fluid were consistent with the effects of OVA sensitization and challenge, and with fish oil intake, in the Bargut et al. study [26].

Mucus hypersecretion contributes to the pathology of asthma [17]. In humans with asthma, there is more mucus in central and peripheral airways caused by both hypertrophy and hyperplasia of airway secretory tissue [17]. Secretagogues produced during inflammation, including cytokines, prostaglandins and leukotrienes, trigger a number of signaling pathways associated with altered Muc gene expression, and result in increased mucin synthesis and secretion (reviewed in [18]). This in turn is associated with goblet cell hyperplasia, airway mucus hypersecretion, and respiratory complications [17].

Mucins are complex glycoproteins having a linear peptide backbone linked to hundreds of carbohydrate side chains [17]. Mucins are divided into two structurally and functionally distinct classes: secreted mucins and membrane-associated mucins. Muc5ac and Muc5b are the predominant messages responsible for the secreted mucins in healthy airway mucus [17]. Muc5ac message is produced by surface goblet-cells. Muc5b is expressed primarily by submucosal glands in humans, but these glands are lacking in mice. In mouse models of asthma, airway epithelial cells show upregulation of Muc5b message in association with mucus-cell hyperplasia (reviewed in [16]). As described in detail previously [16], co-expression of Muc5ac and Muc5b in surface goblet cells is similar to that seen in fetal lung, and suggests that injury may induce some type of progenitor cell proliferation or transdifferentiation of differentiated cells that mimics the behavior of airway epithelial cells in fetal lung development. In healthy lungs, Muc4 is a gene message localized to basal, ciliated, and goblet cells [17]. Cell-tethered mucins such as Muc4 can be shed from the cell surface or directly secreted, but their functional role in mucus is uncertain [19].

Because mucins are large and have complex carbohydrate structures, more data is available on gene regulation at the mRNA level than on the proteins themselves [16]. In our study, all OVA-sensitized and challenged mice had higher mRNA concentrations of Muc5ac, Muc5b, and Muc4 compared with Negative Control mice; however, mice consuming fish oil-enriched diet had even higher concentrations of Muc5b and Muc4 mRNA compared with Positive Control mice. This increase in mucin gene expression, however, was not associated with increased mucus deposition.

It is interesting to speculate why increased lung tissue levels of mucin gene message (Muc4, Muc5ac, and Muc5b) in mice fed fish oil, and to a lesser extent corn oil, are associated with attenuated mucus production compared with Positive Control mice. Many inflammatory mediators and byproducts increase mucin gene expression and are increased in airway sections of patients with asthma. This includes IL-8, a potent proinflamatory mediator and neutrophil chemoattractant. It is known that long-chain (n-3) fatty acids decrease production of inflammatory cytokines, including IL-8 [5]. IL-8 has been shown to regulate mucin gene expression at the posttranscriptional level, increasing transcript stability by altering the levels of RNA-binding proteins [31]. Consequently, sustained MUC gene expression in inflamed airways results in mucus hypersecretion, overproduction, and airway obstruction [31]. Thus, it is possible that if inflammatory mediators are down-regulated by fish oil PUFA, then MUC gene stability is decreased and less mucus is produced even though mucin gene expression is increased. Because our mice fed fish oil-enriched diet had significantly less PAS+ mucus-producing cells compared with Positive Control mice, we agree with Voynow et al. [18] that signaling pathways in lung tissue that regulate mucus producing cells may be different from those that regulate mucin gene expression. Further studies are needed to determine the relationship between mucin gene expression and mucus production, and to determine if the suppressive effects of dietary (n-3) PUFAs on mucus production are direct, or use a pro-inflammatory cytokine-dependent mechanism that alters mucin gene stability.

Inflammatory Th2 cytokines, including IL-4 and IL-13, are most frequently associated with Muc5ac gene expression (reviewed in [16]). Although IL-4 may act indirectly, there is strong evidence that IL-13 acts directly to induce airway epithelial cells to upregulate Muc5ac expression and differentiate into mucous producing cells (reviewed in [16]). In our study, we found that OVA-sensitized and challenged mice, regardless of diet, had increased IL-13 gene expression in lung tissue but decreased IL-13 cytokines levels in BAL fluid compared with Negative Control mice that were sensitized and challenged with saline. Bargut et al. [26] also observed that OVA-sensitized and challenged mice had increased IL-13 cytokine levels in lung tissue compared with Negative Control mice that were sensitized and challenged with saline. It is likely that airway BAL fluid in our study was less reliable than measuring tissue cytokine concentrations. Regardless, we showed that our model was working, as lung tissue levels of proinflammatory cytokine messages (IL-4, IL-5, and IL-13) were all increased by OVA sensitization and challenge. Others have shown that gene expression in lung tissues doesn’t always correlate to protein levels of IL-5 and IL-13 measured by ELISA. For example, protein levels of IL-5 and IL-13 measured in studies using a murine model of OVA-induced allergic lung inflammation have shown different results after fish oil supplementation, both enhancing production of cytokines [32] or attenuating them [26].

Because asthma results from Th2-driven inflammation of airways following sensitization and repeated exposure to an aeroallergen, we also investigated whether recruitment of inflammatory cells around airways was affected by feeding (n-3) PUFA enriched food. Overall, there were significantly less CD45+ inflammatory cells in mice fed fish-oil enriched food compared with Positive Control mice. Cytokines play a critical role in perpetuating and amplifying inflammatory responses in asthma [33]. The cytokines IL-4, IL-5, and IL-13 are derived from Th2 cells and are specific for allergic inflammation. IL-4 is an upstream cytokine that promotes Th2 cell differentiation and enhances the isotype switch to IgE. IL-5 is highly effective for eosinophil recruitment. IL-13 is similar to IL-4 in regulating IgE production, but unlike IL-4 it does not regulate T-cell differentiation into Th2 cells. IL-13 promotes airway hyperreactivity and increases Muc5ac gene expression, protein synthesis, and mucus production in airways [34].

Other researchers have demonstrated a number of mechanisms whereby inflammation might be altered by long-chain, highly polyunsaturated (n-3) FA; although, (n-3) FA dose and composition (e.g., DHA individually versus fish oil) must be taken into consideration [35]. In general, (n-3) FA decrease production of pro-inflammatory eicosanoids, cytokines, and reactive oxygen species [5]. This is, in part, because EPA acts as a negative allosteric effector of Δ-5 desaturase, which is the rate-limiting enzyme in AA metabolism, and competes with AA as the substrate for 5-lipoxygenase in LT production (reviewed in [6]). The (n-3) PUFA also modulate adhesion molecule expression [5, 11]. Individuals with asthma produce significantly greater quantities of LT than nonasthmatic individuals [14]. The dihydroxy LT (LTB_4_) attracts leukocytes by chemotaxis, whereas cysteinyl LT (LTC_4_, LTD_4_, and LTE_4_) are potent bronchoconstrictors that also induce mucus secretion and promote eosinophil chemotaxis. The discovery that BLT1, a G-protein-coupled receptor, is a high affinity receptor specific for LTB_4_ has renewed interest in the role of LTB_4_ in allergen-induced airway hyperresponsiveness and inflammation (reviewed in [36]). Specifically, BLT1-null mice with OVA-induced bronchial asthma did not develop airway hyperresponsiveness or eosinophilic inflammation, and had reduced IgE production and decreased IL-5 and IL-13 in BAL fluid, suggesting an attenuated Th2-type response [37]. Researchers have altered the pathogenesis of chronic allergic airway inflammation using LTC_4_ null mice [38], a 5-lipoxygenase inhibitor [21], or cysteinyl LT receptor antagonism [39]. Also, protectin D1, a natural product of a DHA signaling pathway, displays potent counter-regulatory actions on airway concentrations of proinflammatory peptide and lipid mediators, airway mucus, and leukocyte accumulation, all of which dampen airway inflammation and hyperresponsiveness [40]. Finally, it has also been shown that highly polyunsaturated (n-3) FA inhibit agonist-induced TLR activation [41, 42]. If activation of TLR-4 is suppressed by (n-3) PUFA, then signaling pathways downstream, including the NF-κB pathway, and ensuing cellular responses (pro-inflammatory cytokine production) are suppressed. The (n-3) PUFA are more potent inhibitors of TLR-4 and TLR-2 activation compared with the (n-6) PUFA [41]. Thus, there are a number of mechanisms whereby (n-3) PUFA have the potential to modulate early inflammatory signal transduction processes and reduce inflammation. In addition, OVA sensitization and challenge is not a model of asthma per se, but rather a model of acute allergic airway and pulmonary inflammation. Thus, it is possible that feeding (n-6) and (n-3) FA-enriched foods alters the response to OVA challenge and sensitization.

In conclusion, we demonstrated that at sufficiently high intake (20% of DM), dietary enrichment with either fish oil (n-3) or corn oil (n-6) PUFA attenuates mucus production associated with OVA-induced allergic lung inflammation. However, dietary fish oil-enrichment additionally decreases CD45+ inflammatory cell infiltration. Further studies are needed to determine the relationship between dietary (n-3) or (n-6) PUFA content, mucus production, and the inflammatory response to OVA in lung tissue in order to recommend dietary PUFA supplementation as an option for decreasing the dosage and, consequently, side effects of corticosteroids used to treat asthma.

## Abbreviations

AA: arachidonic acid
ALA: α-linolenic acid
BAL: bronchoalveolar lavage
DHA: docosahexaenoic acid
EPA: eicosapentaenoic acid
FA: fatty acids
LA: linoleic acid
LT: leukotriene
Muc: mucin
MUFA: monounsaturated fatty acids
OVA: ovalbumin
PAS: periodic acid-Schiff
PBS: phosphate buffered saline
PG: prostaglandin
PUFA: polyunsaturated fatty acids
SFA: saturated fatty acids
TBST: Tris buffered saline with Tween
